# Influence of Polishing Protocols on Surface Roughness and Color Properties of a Zirconia-Based Nano-Hybrid Resin Composite

**DOI:** 10.4317/jced.63745

**Published:** 2026-02-26

**Authors:** Eduarda Carla da Silva Souza, Larissa de Jesus Gomes, Leonardo Santos Barros, Débora Alves Nunes Leite Lima, Cecilia Pedroso Turssi, Fabiana Mantovani Gomes França, Roberta Tarkany Basting, Waldemir Francisco Vieira-Junior

**Affiliations:** 1 Faculdade São Leopoldo Mandic, Restorative Dentistry Department, Campinas, São Paulo, Brazil; 2 Universidade Estadual de Campinas, Faculdade de Odontologia de Piracicaba, Restorative Dentistry Department, Piracicaba, São Paulo, Brazil

## Abstract

**Background:**

This study evaluated the impact of polishing procedures on the surface texture and color stability of two nano-hybrid resin composites, with or without zirconia filler.

**Material and Methods:**

Cylindrical specimens (6 × 2 mm) were fabricated using a nano-hybrid resin composite containing zirconia (Forma, Ultradent) or a composite without zirconia (Empress Direct, Ivoclar Vivadent). Specimens were subjected to two finishing and polishing protocols (n = 12): aluminum oxide abrasive disks (Sof-Lex, 3M) or disk-shaped abrasive rubbers (Jiffy, Ultradent). Roughness (Ra, µm) and color (CIEL*a*b* system, CIEDE2000, Vita shade guide units [SGU]) were measured at baseline, after polishing protocol, and after immersion in coffee solution.

**Results:**

were evaluated using linear mixed-effects models for repeated measures, along with Tukey-Kramer, Mann-Whitney, Friedman, and Nemenyi tests ( = 0.05). Results: All groups exhibited an increase in Ra after polishing, with the highest values observed in specimens polished with abrasive rubbers (p &lt; 0.05). SGU values significantly increased for both resin composites following coffee exposure, although the zirconia-based composite showed lower overall SGU changes (p &lt; 0.05). After staining, Eab and E00 values were greater for the abrasive rubber groups compared to the aluminum oxide disk groups and were also higher for the non-zirconia resin composite compared to the zirconia-based material (p &lt; 0.0001).

**Conclusions:**

Polishing with aluminum oxide abrasive disks resulted in smoother surfaces and reduced susceptibility to staining. Nevertheless, the zirconia-containing resin composite demonstrated superior color stability regardless of the polishing method.

## Introduction

The development of restorative materials that exhibit mechanical and aesthetic properties similar to natural teeth remains a challenge in dentistry, particularly in terms of their stability within the oral environment and their clinical longevity (1). Resin composites have been progressively developed and refined over recent decades, becoming excellent materials for direct restorative procedures (2 - 4). Resin composite is a polymeric material composed primarily of filler particles bound to an organic matrix through a coupling agent (5 , 6). This organic matrix typically consists of methacrylate monomers such as Bis-GMA, which is combined with other dimethacrylates, including TEGDMA and UDMA (6 , 7). Filler particles, along with other additives, are incorporated into the polymer matrix to enhance the mechanical performance and clinical durability of resin composites (6). These particles constitute the inorganic content of the composite and may include barium aluminosilicate, silica, zirconia, quartz, ytterbium trifluoride or a combination of different particles (8). Increasing the filler content is directly associated with improved mechanical properties, thereby enhancing the resistance of the composite to challenges imposed by the oral cavity (5). Accordingly, resin-based materials can be classified based on the type, volume and size of filler particles (5 , 8). Furthermore, the morphology and composition of these particles are also important parameters for classification (8 , 9). The characteristics of filler particles directly influence finishing and polishing outcomes. These particles may exhibit varying shapes, such as spherical or irregular (5 , 10). In addition, filler shape and size affect surface wear and properties such as roughness and color stability (5 , 10 - 14). Among these, zirconia [zirconium dioxide] has been widely used in dentistry due to its excellent mechanical strength, biocompatibility, and optical characteristics (15). The incorporation of zirconia can significantly enhance flexural strength, elastic modulus, and fracture resistance in resin composites (16). Thus, commercial resin composites containing zirconia particles have emerged on the market, some of which are marketed by manufacturers as "zirconia nano-hybrid composites." These materials have been scarcely investigated in literature, particularly with respect to color stability and in comparison to commercially available restorative materials without zirconia. Despite the mechanical advantages associated with the incorporation of various filler particles, resin composites can degrade over time, because their surface is exposed to physicochemical processes (1 , 5). Differences in the maintenance of surface polishing characteristics have been observed depending on the type of filler particle (5 , 10). In this context, evaluating the polishing of zirconia-based nano-hybrid resin composites is essential, as the hardness and degree of silanization of the filler particles may affect their polishability and, consequently, the surface roughness and color stability of the restoration. Maintaining low roughness is a factor associated with achieving a greater surface gloss, which can influence color stability, susceptibility to staining, and overall aesthetic properties, as well as biofilm accumulation (18 , 19), surface wear (20 , 21), and the patient's perception of smoothness and comfort (22). Although the roughness and color of the material are primarily determined by its optical and mechanical properties, these characteristics are also influenced by the finishing and polishing procedures employed, as well as the polishing system used (10 , 23). Finishing procedures are clinically performed to remove defects and excesses around restorations (10 , 24), while polishing serves to create a smooth and final glossy surface similar to that of natural tooth (10 , 24). The abrasive or cutting element must be harder than the restorative material to ensure the effectiveness of finishing and polishing systems (14 , 25 , 26). Considering the high hardness of zirconia (27) and its potential limitations in adhesion (28), further studies are warranted to identify the most effective polishing systems for zirconia-containing resin composites and to clarify whether the incorporation of zirconia into the resin matrix affects the final polishing outcome and susceptibility to staining. Although the literature suggests that both the quantity and size of filler particles influence the polish achieved (5 , 29), the type of particle may also affect the polishing outcome through its impact on wear resistance and, consequently, the long-term maintenance of surface smoothness (5 , 11). Thus, it is important to investigate the optical behavior, surface roughness retention, and polishability of the resin composite containing zirconia as a filler, as well as to compare these properties with those of composites formulated with different filler compositions. Accordingly, the present study evaluated the surface roughness and color properties of nano-hybrid resin composites with and without zirconia particles after exposure to different polishing procedures. The null hypotheses were: 1- the type of resin composite, when polished with different systems, would not affect surface roughness or color properties; and 2- the polishing protocol would not influence the surface roughness or color stability of resin composites subjected to a coffee staining procedure.

## Material and Methods

- Study Design This study followed a 2 × 2 × 3 factorial scheme, with three independent variables: I) Resin composite: zirconia-based nano-hybrid (Forma, Ultradent, South Jordan, UT, USA) and conventional nano-hybrid (Empress Direct, Ivoclar Vivadent, Schaan, Liechtenstein); II) Finishing and polishing system: abrasive disks (Sof-Lex, 3M/Solventum, St. Paul, MN, USA) and abrasive rubbers (Jiffy, Ultradent, South Jordan, UT, USA); III) Evaluation time point: baseline, after polishing procedure, and after exposure to coffee solution. The experimental units consisted of 48 disk-shaped specimens fabricated from the two resin composites tested (n = 12). The analyses included surface roughness (Ra, µm), CIEL*a*b* coordinates (L*, a* and b*), shade guide units (SGU, VITA Classical), and overall color changes (Eab and E00). - Specimen Preparation Each specimen was fabricated by placing the designated resin composite from each group (n=12) into a silicone mold with a 6 mm diameter and a 2 mm thickness using a spatula (LM-Arte Applica, Quinelato, Rio Claro, SP, Brazil). The surface was then covered with a Mylar strip and a glass slide, and an axial load of 500 g was applied for 20 seconds to ensure compaction and elimination of air bubbles. Light curing was performed using an LED unit (Valo, Ultradent, South Jordan, UT, USA) in standard mode (1000 mW/cm²), for 20 seconds. The classification and composition of the resin composites used are described in Table 1.

Table 1

- Finishing and Polishing Protocol The specimens from each group were randomly divided into two subgroups, which received different finishing and polishing treatments. The systems used are described in Table 2.

Table 2

All procedures were performed using a bench-top electric motor (Electromatic MS, KaVo, Biberach, Germany). The polishing instruments were replaced after use on three specimens. Specimens in the abrasive disk group were finished and polished using Sof-Lex disks (3M/Solventum, St. Paul, MN, USA) in a sequence of grit sizes (coarse, medium, fine, and superfine), each applied for 10 seconds at 10,000 rpm under water cooling. Afterward, the specimens were rinsed with distilled water for 10 seconds, cleaned in an ultrasonic bath (Thorton, Vinhedo, SP, Brazil) for 10 minutes, dried, and stored in relative humidity. Specimens in the abrasive rubber group underwent the same procedure using a sequence of disk-shaped abrasive rubbers (Jiffy, Ultradent, South Jordan, UT, USA), based on grit size (green, yellow, and white), also applied for 10 seconds each at 10,000 rpm under water cooling. After polishing, the samples were likewise rinsed with distilled water for 10 seconds, cleaned in an ultrasonic bath, dried, and stored in relative humidity (30). - Surface Roughness Evaluation Surface roughness was measured using a surface profilometer (Surftest SJ-210, Mitutoyo Co., Kanagawa, Japan) at three time points: at baseline, after polishing procedure, and after immersion in the coffee solution. Average surface roughness (Ra, µm) was calculated considering 1.25 mm of stylus path, 0.25 of cut-off, and a speed of 0.25 mm/s. Measurements were performed in triplicate and the mean was considered the final Ra value, with the stylus traversing the geometric center of each sample in different directions. - Color analyses Color properties were determined using a digital spectrophotometer (VITA Easyshade, VITA Zahnfabrik, Bad Sackingen, Germany) under standardized light conditions at baseline, after polishing, and after coffee immersion. The VITA Easyshade spectrophotometer was calibrated according to the manufacturer's instructions, and readings were recorded using CIEL*a*b coordinates. Color differences between time points were calculated using the Eab and CIEDE2000 (E00) (31).

(1) $${\Delta E}_{*ab}=\sqrt{{\left(\Delta L*\right)}^{2}+{\left(\Delta a*\right)}^{2}+{\left(\Delta b*\right)}^{2}}$$

(2) $${\Delta E}_{00}=\sqrt{{\left(\frac{\Delta L\prime }{kS_{L}}\right)}^{2}+{\left(\frac{\Delta C\prime }{kS_{C}}\right)}^{2}+{\left(\frac{\Delta H\prime }{kS_{H}}\right)}^{2}+R_{T}\left(\frac{\Delta C\prime }{kS_{C}}\right)\left(\frac{\Delta H\prime }{kS_{H}}\right)}$$

Additionally, each specimen was assessed using the VITA Classical scale (SGU), scored from 1 to 16 based on luminosity, where 1 (B1) indicated the highest luminosity and 16 (C4) the lowest (32). - Coffee Immersion Protocol After polishing procedures, the samples were individually immersed in coffee solution (5 ml). The solution was prepared by adding 4 g of coffee powder (Nestlé, São Paulo, SP, Brazil) to 300 ml of previously boiled water, then steeped for 5 minutes (33). Specimens were immersed immediately after preparation and stored at 37°C for 7 days (34), during which the coffee solution was replaced daily. After staining protocol, specimens were rinsed in an ultrasonic bath with distilled water for 10 minutes, dried, and re-evaluated for final color and surface roughness. - Statistical Analysis Statistical analyses were performed using the R program (R Core Team, Vienna, Austria), considering a significance level of 5%. Exploratory analyses were performed to define the appropriate statistical tests for each variable. Linear mixed-effects models for repeated measures and the Tukey-Kramer test were used for the L* and b* coordinates. The remaining variables were analyzed using the Mann-Whitney test for comparisons between resin composites and polishing methods, and the Friedman and Nemenyi tests for comparisons over time.

## Results

The p-values for the main effects and interactions are presented for each variable in the corresponding tables. - Roughness As shown in Table 3, both resin composites exhibited a significant increase in surface roughness after polishing.

Table 3

Following polishing and staining with coffee, roughness values were significantly higher in specimens polished with abrasive rubbers compared to those polished with abrasive disks, regardless of resin composite. No significant difference in roughness was observed between the two resin composites. - Color (L*, a*, and b* coordinates) As shown in Table 4, both resin composites exhibited a significant increase in the L* coordinate value after polishing with the abrasive disk.

Table 4

After staining with coffee, L* values decreased for both composites, regardless of the polishing system. The zirconia-based resin composite showed higher L* values than the conventional resin composite at all time points. Table 5 presents the a* coordinate results.

Table 5

A significant increase in a* values was observed after polishing and after staining in all groups. The zirconia-based resin composite showed higher a* values after polishing with abrasive rubbers than with abrasive disks. In contrast, the conventional resin composite showed higher a* values after staining when polished with abrasive rubbers compared to abrasive disks. At all evaluation time points, the conventional resin composite exhibited higher a* values than the zirconia-based composite. As shown in Table 6, b* values increased significantly after staining with coffee in all groups.

Table 6

**Figure 1:**
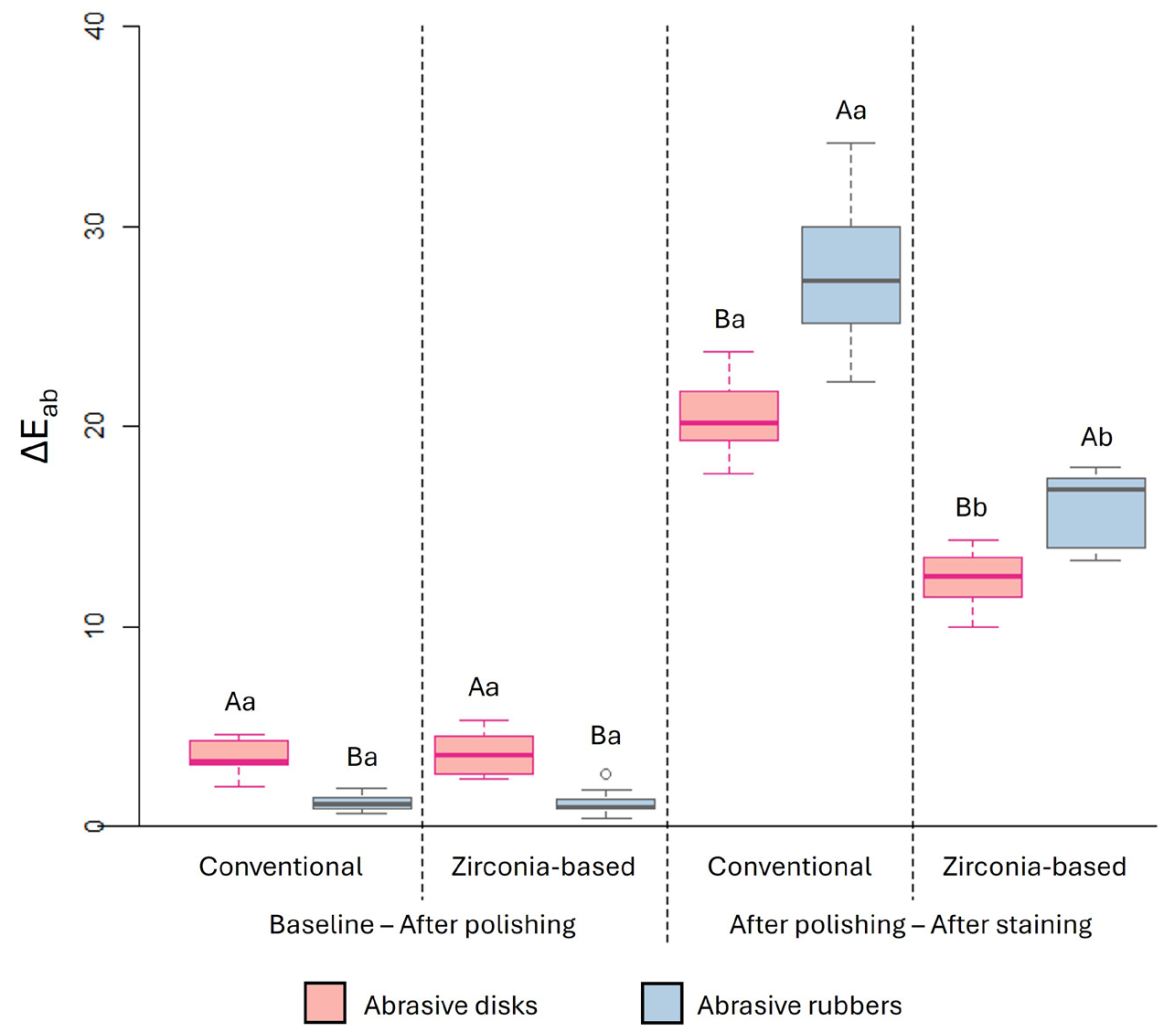
Boxplot of the overall color change (ΔEab) as a function of resin composite, polishing system and time.Caption: Different letters indicate statistically significant differences between polishing systems (uppercase) and resin composites (lowercase) within the same time period (p ≤ 0.05). Differences between the polishers: p &lt; 0.0001; Differences between the resin composites at coffee exposure: p &lt; 0.0001; Absence of differences between the resin composites at polishing time: p = 0.8625 for abrasive disks, and p = 0.5834 for abrasive rubbers.

**Figure 2:**
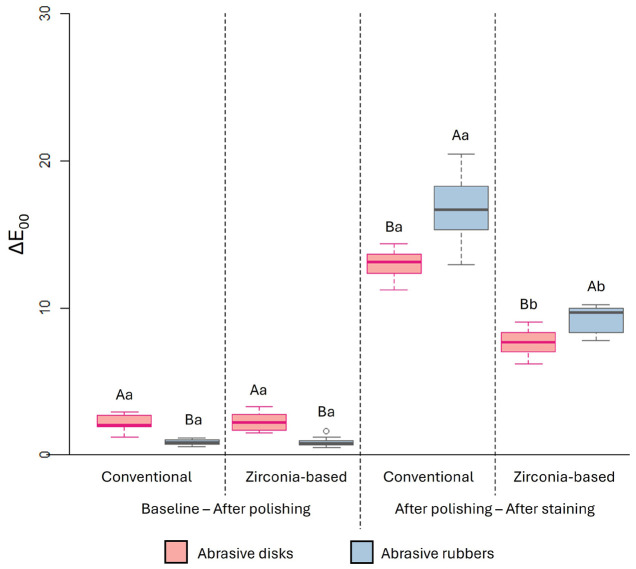
Boxplot of the overall color change (ΔE00) as a function of resin composite, polishing system and time.Caption: Different letters indicate statistically significant differences between polishing systems (uppercase) and resin composites (lowercase) within the same time period (p ≤ 0.05). Differences between the polishers: p &lt; 0.0001; Differences between the resin composites at coffee exposure: p &lt; 0.0001; Absence of differences between the resin composites at polishing time: p = 0.9081 for abrasive disks, and p = 0.5637 for abrasive rubbers.

Following staining, both resin composites exhibited higher b* values when polished with abrasive rubbers. In all conditions, the conventional resin composite showed higher b* values than the zirconia-based composite. - Color Alteration (Eab and E00) The results for color alteration are shown in Figure 1 (Eab) and Figure 2 (E00).

1

2

After polishing, both resin composites exhibited higher Eab and E00 values when polished with abrasive disks compared to abrasive rubbers. Considering baseline vs. polishing procedure, no significant difference in Eab and E00 was observed between the resin composites. However, after staining with coffee compared to the post-polishing condition, Eab and E00 values were significantly higher for the conventional resin composite than for the zirconia-based composite and were higher in the groups polished with abrasive rubbers. - Color (SGU, VITA Scale) As shown in Table 7, SGU scores increased significantly after staining with coffee in all groups, regardless of resin composite or polishing method.

Table 7

After polishing, the conventional resin composite exhibited higher SGU values when polished with abrasive rubbers than with abrasive disks. SGU readings for the zirconia-based resin composite were higher after staining in specimens polished with abrasive rubbers. In all situations, the conventional resin composite showed higher SGU scores than the zirconia-based composite.

## Discussion

The maintenance of color, gloss, and surface polish is essential for the clinical performance of resin composite restorations (10 , 35 , 36). The loss of these characteristics may lead to restoration replacement, which can damage the tooth by enlarging the cavity and consequently removing sound structure (37 , 38). Both polishing and the characteristics of filler particles are associated with the mechanical resistance and staining behavior of resin composites (5). Based on the results obtained, both null hypotheses were rejected, since the type of resin composite and the polishing system affected surface roughness and color properties. Moreover, immersion in coffee solution led to additional degradation in both parameters. Surface roughness is considered a clinically important parameter, since it determines the ability of the surface to retain biofilm and pigments (18 , 39). Filler particle shape has been suggested to influence surface roughness (40), with irregular particles generally associated with rougher surfaces. Although the resin composites varied in composition, they exhibited comparable surface roughness values. This may be attributed to similarities in filler volume percentage, classification as nano-hybrid composites, and average particle size, as shown in Table 1. In contrast, the polishing system significantly affected roughness in both materials, with the abrasive rubbers resulting in higher Ra values, exceeding the clinically acceptable threshold of 0.2 µm (41). In general, lower Ra values are achieved with aluminum oxide-impregnated disks (10 , 42), and the characteristics of the polishing abrasive particles can affect the contact surface (43). Considering the Mohs hardness scale: diamond &gt; silicon carbide &gt; tungsten carbide &gt; aluminum oxide &gt; zirconium dioxide (44 , 45), it is possible that the higher hardness of silica particles in the abrasive rubbers led to the dislodgement of filler particles or scratching of the superficial, resin-rich layer (46 , 47). Additionally, the abrasive disks involved four grit levels, compared to three in the abrasive rubber group, a difference that, along with longer application times and additional polishing steps, has been associated with more favorable roughness outcomes (24). Finally, although the abrasive rubber particles may be smaller (Table 2), abrasive disks distribute particles homogeneously across their surface, while rubbers retain abrasives within a flexible matrix. Future studies should employ microscopic imaging to characterize these instruments more precisely. Moreover, it is important to emphasize that various finishing and polishing protocols are available, as well as polishing systems with different abrasive formulations (12 , 35). The present study aimed to differentiate the polishing system types (rubbers versus disks) by applying them in a sequence of decreasing abrasiveness, in accordance with previous studies (13 , 22 , 38). The roughness data help explain the color change patterns observed, namely higher Eab and E00 values after polishing with disks (baseline vs. post-polishing), and higher values after staining in the rubber groups (post-polishing vs. post-staining). Roughness and color are correlated properties (33), and smoother surfaces produced by disks reflect more light, resulting in greater changes in the L* coordinate (towards white) and Eab and E00 values exceeding the proposed acceptability thresholds (48) for color change (12). Conversely, rougher surfaces polished with abrasive rubbers retained more pigments, contributing to decreases in L* (toward black), and increases in a* (toward red) and b* (toward yellow), resulting in more pronounced Eab and E00 values after coffee immersion. Coffee beverage was selected as the pigmentation agent due to its widespread consumption and well-documented staining capacity (49 , 50). Its yellow pigments have polarity and affinity for the polymer matrix (51), which likely explains the observed increases in the b* coordinate. However, because coffee has a pH near 5, it does not significantly alter surface roughness (12), consistent with the present findings. After coffee immersion, both composites exhibited clinically perceptible color changes (Eab &gt; 1.2; E00 &gt; 0.8), and surpassed the acceptability thresholds (Eab &gt; 2.7; E00 &gt; 1.8) (48), regardless of the polishing system. Such changes may stem from intrinsic factors (e.g., material composition) and extrinsic ones (e.g., pigment absorption from staining agents) (34 , 52). In this context, the zirconia-based resin composite was less susceptible to staining than the conventional resin composite. Although polishing affects stain susceptibility, the formulation of the composite also plays a central role (12 , 43). As a crystalline oxide of zirconium-based material, zirconia contains a polycrystalline structure that may form a substrate less prone to pigment uptake (53 , 54). The zirconia-based composite tested in this study demonstrated clinically acceptable color stability and resistance to degradation under other conditions (55 , 56). The incorporation of zirconia particles may influence the overall surface microhardness of resin composites, as a previous study reported higher microhardness values for the Forma compared with Empress (57). However, the present findings indicate that this does not adversely affect the polishability of the material, whereas both resin composites exhibited similar patterns of surface roughness alteration. Notably, the resin composite containing zirconia in its formulation demonstrated more favorable color stability. Nonetheless, as inherent to in vitro experimental models, the present study has certain limitations. Although the two composites exhibit similarities in monomer matrix composition and filler volume fractions, they are manufactured by different companies, which alone may account for some of the differences observed. Moreover, while the findings are of interest, it is not possible to isolate the specific contribution of zirconia, since the resin matrix composition also plays a critical role in determining the properties of resin-based materials. The chemical nature of the monomers used in the resin matrix is known to influence color stability and related properties, such as water sorption and solubility (58). In addition, other factors may affect material performance, including the hardness mismatch between filler and matrix, the degree of monomer conversion, and the quality of the silane coupling at the filler-matrix interface (40). Further investigations using experimental composites with controlled formulations of individual components, as well as studies including a broader range of commercial materials from different manufacturers, are recommended.

## Conclusions

Zirconia-based resin composite was less susceptible to staining by coffee than conventional resin composite, which did not contain zirconia. Additionally, the finishing and polishing system employing abrasive disks resulted in improved smoothness and better color stability for both types of resin composites.

## Figures and Tables

**Table 1:** Restorative materials and composition according to manufacturers.

Resin Composite(Manufacturer)	Shade	Organic Matrix Composition	Classification/Filler Particle Composition
Empress Direct(Ivoclar Vivadent, Schaan, Liechtenstein)	A1E	Bis-GMA, UDMA, cycloaliphatic dimethacrylate, propoxylated bisphenol-A dimethacrylate	Conventional nano-hybrid Average filler size: 150 nm – 0.7 µm Barium glass, ytterbium trifluoride, mixed oxides, silicon dioxide 78.1 wt% and 66 vol%
Forma (Ultradent, South Jordan, UT, USA)	A1E	BisGMA, TEGDMA, Bis-EMA, UDMA	Zirconia-based nano-hybrid Average filler size: 5 – 50 nm nm Zirconia/silica particles, barium glass 64.8 vol%

Abbreviations: Bis-GMA, bisphenol A glycidyl methacrylate; UDMA, urethane dimethacrylate; TEGDMA, triethylene glycol dimethacrylate; Bis-EMA, ethoxylated bisphenol dimethacrylate; EDMAB, ethyl 4-dimethylaminobenzoate. Information extracted from manufacturer labels.

**Table 2:** Manufacturers and composition of the finishing and polishing systems used.

Finishing and Polishing System	Description	Manufacturer	Composition
Sof-Lex	Abrasive disk	3M/Solventum (St. Paul, MN, USA)	Polyester disks impregnated with aluminum oxide: coarse, medium, fine, and superfine
Jiffy	Disk-shaped rubber system	Ultradent (South Jordan, UT, USA)	Rubber polishers impregnated with silicon and aluminum oxide particles: coarse (green), medium (yellow), and fine (white)

Information extracted from manufacturer labels.

**Table 3:** Median (minimum and maximum values) of roughness (Ra, µm) according to resin composite, polishing system, and time point.

Resin Composite	Polishing System	Time Point			p-value
		Baseline	After Polishing	After Staining	
Conventional	Abrasive disks	0.043 (0.030; 0.101) Ba	0.153 (0.060; 0.261) Ab	0.140 (0.055; 0.225) Ab	0.0038
	Abrasive rubbers	0.042 (0.030; 0.104) Ba	0.298 (0.056; 0.747) Aa	0.297 (0.117; 0.763) Aa	<0.0001
p-value		0.9310	0.0051	0.0012	
Zirconia-based	Abrasive disks	0.042 (0.027; 0.145) Ba	0.160 (0.085; 0.237) Ab	0.154 (0.089; 0.255) Ab	0.0003
	Abrasive rubbers	0.045 (0.028; 0.133) Ba	0.311 (0.181; 0.612) Aa	0.274 (0.080; 0.551) Aa	<0.0001
p-value		0.6033	<0.0001	0.0017	

Distinct letters indicate statistically significant differences (p ≤ 0.05): uppercase letters for comparisons across rows (polishing systems) and lowercase letters for comparisons down columns (evaluation time point).

**Table 4:** Mean (standard deviation) of L* values according to resin composite, polishing system, and time point.

Resin Composite	Polishing System	Time Point		
		Baseline	After Polishing	After Staining
Conventional	Abrasive disks	88.73 (1.01) Ba	92.11 (1.37) Aa	74.50 (1.82) Ca
	Abrasive rubbers	88.31 (0.79) Aa	89.01 (0.87) Ab	68.06 (2.92) Bb
Zirconia-based	Abrasive disks	*90.65 (0.93) Ba	*94.21 (1.05) Aa	*85.13 (1.46) Ca
	Abrasive rubbers	*90.40 (1.12) Aa	*90.93 (1.09) Ab	*81.08 (1.40) Bb

*Significantly different from the conventional resin composite under the same polishing condition and time (p ≤ 0.05). Distinct letters (uppercase across rows and lowercase within columns) indicate statistically significant differences (p ≤ 0.05). p(resin composite) < 0.0001; p(polishing system) < 0.0001; p(resin composite × polishing system) = 0.2221; p(time) < 0.0001; p(resin composite × time) < 0.0001; p(polishing system × time) < 0.0001; p(resin composite × polishing system × time) = 0.0072.

**Table 5:** Median (minimum and maximum values) of a* values according to resin composite, polishing system, and time point.

Resin Composite	Polishing System	Time Point			p-value
		Baseline	After Polishing	After Staining	
Conventional	Abrasive disks	0.80 (0.00; 1.20) Ca	1.00 (0.20; 1.50) Ba	5.80 (5.40; 7.10) Ab	<0.0001
	Abrasive rubbers	0.65 (0.00; 1.10) Ca	1.30 (0.70; 1.50) Ba	7.55 (6.20; 9.40) Aa	<0.0001
p-value		0.3865	0.1333	0.0003	
Zirconia-based	Abrasive disks	*-0.20 (-0.50; 0.10) Ca	*0.20 (-0.10; 0.30) Bb	*3.25 (2.60; 4.20) Aa	<0.0001
	Abrasive rubbers	*-0.05 (-0.40; 0.10) Ca	*0.40 (0.10; 0.60) Ba	*3.65 (3.00; 4.20) Aa	<0.0001
p-value		0.3263	0.0056	0.1749	

*Significantly different from the conventional resin composite under the same polishing condition and time (p ≤ 0.05). Distinct letters (uppercase across rows and lowercase within columns) indicate statistically significant differences (p ≤ 0.05).

**Table 6:** Mean (standard deviation) of b* values according to resin composite, polishing system, and time point.

Resin composite	Polishing system	Time Point		
		Baseline	After Polishing	After Staining
Conventional	Abrasive disks	23.34 (0.66) Ba	22.88 (0.72) Ba	31.74 (1.98) Ab
	Abrasive rubbers	23.32 (0.55) Ba	22.78 (0.47) Ba	39.43 (2.88) Aa
Zirconia-based	Abrasive disks	*16.96 (0.94) Ba	16.59 (0.74) Ba	*24.30 (0.71) Ab
	Abrasive rubbers	*16.74 (0.59) Ba	16.03 (0.50) Ba	*28.02 (1.66) Aa

*Significantly different from the conventional resin composite under the same polishing condition and time (p ≤ 0.05). Distinct letters (uppercase across rows and lowercase within columns) indicate statistically significant differences (p ≤ 0.05). p(resin composite) < 0.0001; p(polishing system) < 0.0001; p(resin composite × polishing system) = 0.0013; p(time) < 0.0001; p(resin composite × time) < 0.0001; p(polishing system × time) < 0.0001; p(resin composite × polishing system × time) = 0.0003.

**Table 7:** Median (minimum and maximum values) of the VITA scale shade guide units (SGU) according to resin composite, polishing system, and time point.

Resin Composite	Polishing System	Time Point			p-value
		Baseline	After Polishing	After Staining	
Conventional	Abrasive disks	3 (3; 5) Ba	3 (3; 3) Bb	15 (15; 15) Aa	0.0001
	Abrasive rubbers	4 (3; 5) Ba	5 (3; 5) Ba	15 (15; 16) Aa	<0.0001
p-value		0.2987	<0.0001	0.0833	
Zirconia-based	Abrasive disks	*2 (2; 2) Ba	*2 (2; 2) Ba	*9 (2; 11) Ab	0.0063
	Abrasive rubbers	*2 (2; 2) Ba	*2 (2; 2) Ba	*11 (11; 11) Aa	0.0001
p-value		1.000	1.000	0.0153	

*Significantly different from the conventional resin composite under the same polishing condition and time (p ≤ 0.05). Distinct letters (uppercase across rows and lowercase within columns) indicate statistically significant differences (p ≤ 0.05).
